# Editorial: Ambient ionization mass spectrometry: From fundamentals to real-life applications

**DOI:** 10.3389/fchem.2023.1182894

**Published:** 2023-03-30

**Authors:** Haixing Wang, Pui-Kin So, Ahsan Habib, Yu Xu, Federica Bianchi

**Affiliations:** ^1^ Key Laboratory of Drug Monitoring and Control of Zhejiang Province, National Anti-Drug Laboratory Zhejiang Regional Center, Hangzhou, China; ^2^ University Research Facility in Life Sciences, Hong Kong Polytechnic University, Kowloon, Hong Kong SAR, China; ^3^ Department of Chemistry, University of Dhaka, Dhaka, Bangladesh; ^4^ Department of Chemistry, Life Science and Environmental Sustainability, University of Parma, Parma, Italy

**Keywords:** ambient ionization mass spectrometry (AIMS), desorption electrospray ionization (DESI), paper spray ionization (PSI), wooden-tip (WT), direct analysis in real-time (DART)

Mass spectrometry (MS) is a powerful analytical technique contributing towards both fundamental research and real-life applications thanks to its high specificity and sensitivity. Many MS methods coupled with different separation techniques such as gas- and liquid-chromatography have been proposed for the analysis of complex matrices. However, the labor-intensive and time-consuming sample pretreatment required prior to MS analysis could dampen the application of this technique. The development of ambient ionization MS (AIMS) has paved the way for the direct MS analysis of complex samples with little or no sample pretreatment under ambient conditions, thus reducing analysis time and cost. The first AIMS technique, i.e., desorption electrospray ionization (DESI) was proposed in 2004 by Takáts et al. (2004). After that, different other techniques including electrospray ionization with paper, wooden-tip, laser ablation, and thin-layer chromatography (TLC) plate, rapid evaporative ionization with surgical diathermy, and direct analysis in real-time (DART) were developed and widely applied in life, food, environmental and forensic sciences (Nemes and Vertes, 2007; Yew et al., 2008; Schäfer et al., 2009; Shrestha and Vertes, 2009; Wang et al., 2010; Cheng et al., 2011; Hu et al., 2011; Bianchi et al., 2019). This Research Topic was planned to encourage original research about the fundamentals and applications of AIMS techniques.

Paper spray ionization mass spectrometry (PSI-MS) is an important AIMS technique. However, the mechanism of PSI still needs to be studied. Nguyen et al. have thoroughly assessed certain significant variables that may have an impact on the ionization efficiency of PSI, including electric field, solvent supply rate, and paper properties. With the rise in electric voltage, it was found that the single cone-jet, multi-jet, and rim-jet were all accurately characterized as paper spray modes. As we all know, AIMS can reduce the time for sample purification and extraction. With the help of DART-MS analysis after dispersive solid phase extraction (QuEChERS), Tata et al. successfully screened 40 toxicants including carbamates, organophosphate and chlorinated pesticides, coumarins, metaldehyde, and strychnine in poison baits and autopsy specimens.

Yang et al. achieved the auto-sampling concept by using paper strips as adsorbents to capture tobacco smoke and then directly analyzing through coupling with the MS system. The essential components of auto-sampling were optimized using nicotine, a typical constituent of tobacco smoke. The manufacturers of tobacco smoke from various sources were also looked into, and different varieties of tobacco smoke were compared using multivariate variable analysis. A single tobacco sample can be directly sampled and analyzed using this method in a matter of minutes. The overall findings demonstrate the potential of PSI-MS as a potent technique for integrating analyte Research Topic, extraction, ionization, and identification in volatile matrices, particularly smoke.

Wang et al. developed a wooden-tip electrospray ionization-mass spectrometry-based method for the determination of nicotine and cotinine in meconium samples, which is commonly performed for the evaluation of nicotine exposure in the infant during pregnancy in clinics. By optimizing various experimental conditions, e.g., choice of spraying/extraction solvent, Wang et al. demonstrated that WT-ESI-MS allowed rapid determination of nicotine and cotinine in raw meconium samples with good sensitivity, specificity, reproducibility, and accuracy. With the desired analytical performance and the features of simple, rapid, and low cost, this study highlighted the potential application of WT-ESI-MS in daily point-of-care analysis.

An AMS technique has been proposed by Liu et al. for the high-throughput analysis of biological tissues. Wooden-tip electrospray ionization together with high-resolution mass spectrometry followed by multivariate data analysis was presented as a valuable tool for the rapid diagnosis of thyroid cancer allowing for marker discovery together with a better understanding of the molecular composition of the investigated tissues.

The great potential of AIMS has been demonstrated in different research fields: from food to environmental analysis, from clinical to forensics/toxicological applications, allowing for high-throughput screening and reduced sample handling. In the next future, the development of novel sample substrates able to improve both the ionization of the investigated analytes and the reliability of quantitative analyses will be the key Research Topic in this field. Chemometrics will also play a pivotal role in handling the great amount of complex data produced by AIMS techniques with the final aim of achieving a comprehensive evaluation of the system under investigation.
